# Roles of FGF20 in dopaminergic neurons and Parkinson's disease

**DOI:** 10.3389/fnmol.2013.00015

**Published:** 2013-05-31

**Authors:** Nobuyuki Itoh, Hiroya Ohta

**Affiliations:** Department of Genetic Biochemistry, Kyoto University Graduate School of Pharmaceutical SciencesKyoto, Japan

**Keywords:** dopaminergic neurons, Fgf, Fgf20, Parkinson's disease, stem cells, SNP

## Abstract

The fibroblast growth factor (FGF) family comprises 22 members with diverse functions in development and metabolism. *Fgf20* was originally identified as a new *Fgf* preferentially expressed in the substantia nigra pars compacta (SNpc). Fgf20, which acts on proximal cells, significantly enhanced the survival of cultured dopaminergic neurons by activating the mitogen-activated protein kinase (MAPK) pathway through Fgf receptor 1c. In the rat model of Parkinson's disease, Fgf20 afforded significant protection against the loss of dopaminergic neurons. The significant correlation of Parkinson's disease with single-nucleotide polymorphisms in *FGF20* indicates that the genetic variability of *FGF20* can be a Parkinson's disease risk. Neural and embryonic stem (ES) cells have been considered as cell resources for restorative transplantation strategies in Parkinson's disease. Fgf20 promoted the differentiation of these stem cells into dopaminergic neurons, which attenuated neurological symptoms in animal models of Parkinson's disease. These findings indicate the importance of FGF20 for the differentiation and survival of dopaminergic neurons and the etiology and therapy of Parkinson's disease.

## Introduction

Fibroblast growth factors (FGFs) are polypeptides with diverse functions in development, metabolism, and neural activities. The FGF family comprises 22 members, which have been classified as paracrine, endocrine, and intracrine FGFs by their mechanisms of action. Most FGFs are paracrine FGFs that act as local signaling molecules (Itoh and Ornitz, 2011). *Fgf20* was originally identified as a new *Fgf* preferentially expressed in the substantia nigra pars compacta (SNpc). Fgf20, a paracrine Fgf, with neurotrophic activity in cultured dopaminergic neurons (Ohmachi et al., 2000, 2003) has been suggested to play important roles in the development of dopaminergic neurons (Grothe et al., 2004; Takagi et al., 2005; Correia et al., 2007; Shimada et al., 2009). In addition, *FGF20* mutations may result in Parkinson's disease (van der Walt et al., 2004; Satake et al., 2007; IPDGC, 2011; Pan et al., 2012; Pihlstrøm et al., 2013; Wang et al., 2013). As these findings indicate that FGF20 may provide useful clues on the etiology and therapy of Parkinson's disease, a succinct review on the roles of FGF20 in dopaminergic neurons and Parkinson's disease has been provided. In this review, we refer to the human and rodent orthologs as *FGF20* and *Fgf20* according to the Human Genome Organization and the Mouse Genome Informatics, respectively.

## Identification of FGF20

**Figure 1:**
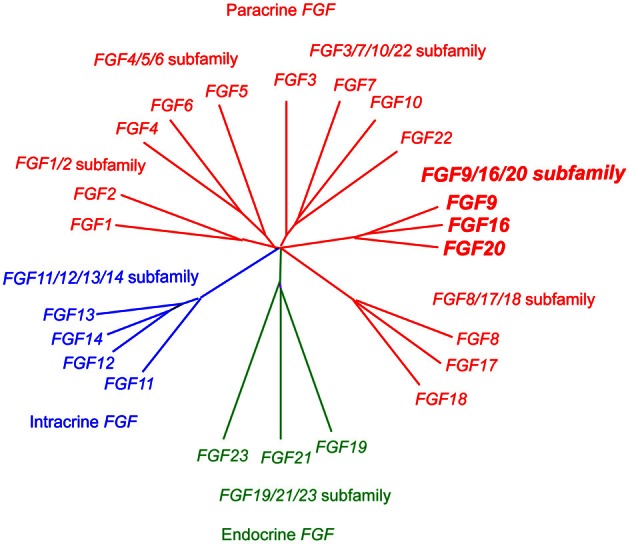
**Evolutionary relationships within the human *FGF* gene family by phylogenetic analysis.** Phylogenetic analysis suggests that 22 *FGF* genes can be arranged into seven subfamilies containing two to four members each. Branch lengths are proportional to the evolutionary distance between each gene.

*Fgf20*, originally identified in the rat brain, encodes a secreted protein of 212 amino acids (Ohmachi et al., 2000). The *FGF* gene family comprising 22 members has been classified into 7 subfamilies; *FGF/1/2, FGF4/5/6, FGF3/7/10/22, FGF8/17/18, FGF9/16/20, FGF11/12/13/14*, and *FGF19/21/23*. FGF20 is a member of the FGF9/16/20 subfamily, which is a paracrine Fgf (Figure 1) (Itoh and Ornitz, 2011).

## FGF20 in dopaminergic neuron survival

As paracrine FGFs are local signal molecules, Fgf20 is expected to act on dopaminergic neurons in the SNpc in a paracrine manner. However, as both Fgf20 and Fgfr1 are expressed in most dopaminergic neurons in the SNpc, Fgf20 may act on them in an autocrine/paracrine manner. Fgf20 was shown to significantly enhance the survival of cultured rat dopaminergic neurons (Ohmachi et al., 2000, 2003).

Paracrine FGF signaling is mediated by the activation of FGFR. Paracrine FGFs bind to FGFRs and induce the phosphorylation of specific cytoplasmic tyrosine residues, which triggers the activation of cytoplasmic signal transduction pathways. Major FGF/FGFR-dependent signaling was shown to be mediated by the mitogen-activated protein kinase (MAPK) and phospholipase-Cγ pathways (Thisse and Thisse, 2005).

**Figure 2:**
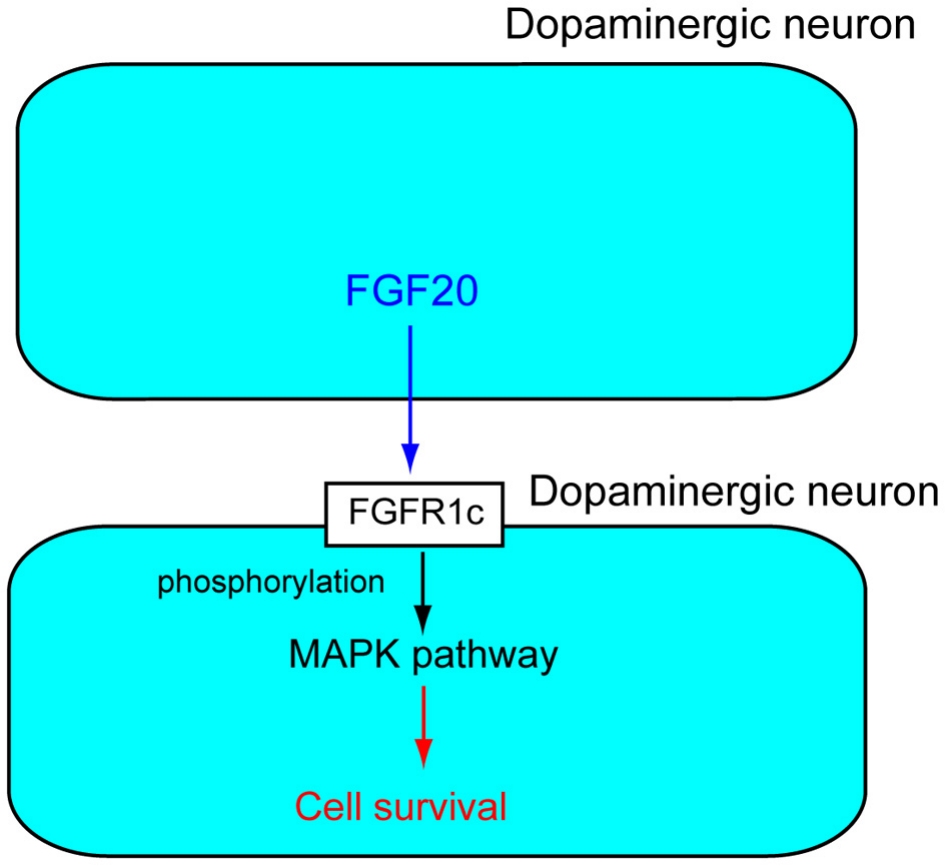
**Action mechanism of FGF20 on dopaminergic neurons.** FGF20 acts on dopaminergic neurons in a paracrine manner. FGF20 binds to FGFR1c and the phosphorylation of specific cytoplasmic tyrosine residues. The phosphorylation of FGFR1c triggers activation of the MPAK pathway, which plays important roles in the survival of dopaminergic neurons.

FGFRs are receptor tyrosine kinases with an extracellular ligand-binding domain, which comprises three immunoglobulin-like domains (I, II, and III). There are seven major FGFR proteins including FGFRs1b, 1c, 2b, 2c, 3b, 3c, and 4, which are generated from four functional *FGFR* genes, *FGFR1*–*FGFR4* by alternative splicing (Zhang et al., 2006). *Fgfr1c* is abundantly expressed in dopaminergic neurons in the SNpc. Fgf20 binds to Fgfr1c with high affinity. Experiments with the Fgfr inhibitor SU5402 or MAPK pathway inhibitor PD98059 indicate that activation of the MAPK pathway by Fgf20 through Fgfr1c is essential for the survival of dopaminergic neurons in the SNpc (Figure 2) (Ohmachi et al., 2003).

Calbindin-negative dopaminergic neurons are preferentially lost in Parkinson's disease. Fgf20 almost completely rescued rat calbindin-negative midbrain dopaminergic neurons from the toxicity of 6-hydroxyldopamine and stress-induced cytosolic dopamine, and promoted dopamine release in calbindin-negative dopaminergic neurons by activating Fgfr1 followed by its downstream cascade activation. These results show that Fgf20 protects the specific midbrain neuron type at most risk in Parkinson's patients (Murase and McKay, 2006).

In the unilateral, 6-hydroxydopamine lesion rat model of Parkinson's disease, supranigral infusion of Fgf20 afforded significant protection against the loss of dopaminergic neurons in the SNpc and striatum. Protection of the nigrostriatal tract was accompanied by the significant preservation of gross locomotion and fine motor movements and the reversal of apomorphine-induced contraversive rotations. These results imply the potential neuroprotective role of Fgf20 in this disease (Sleeman et al., 2012).

## FGF20 in parkinson's disease

Parkinson's disease is a common neurodegenerative disorder. The inability to control movement in patients with this disease has been attributed to the severe loss of dopaminergic neurons within the substantia nigra. Environmental and genetic sources act together in the disease cascade.

*FGF20* has been mapped to 8p21.3–8p22, which is within an area of Parkinson's disease linkage. To test whether *FGF20* genetic variability was a risk factor for Parkinson's disease, five single-nucleotide polymorphisms (SNPs) lying in *FGF20* were examined in a large family study. The highly significant correlation of Parkinson's disease with one SNP located in the intron and two SNPs in the 3′ regulatory region was revealed, which indicated that *Fgf20* genetic variability is a risk factor for Parkinson's disease (van der Walt et al., 2004). In addition, *FGF20* genetic variability was shown to be a risk factor for Parkinson's disease in Japanese and Chinese populations (Satake et al., 2007; Pan et al., 2012), while, was not a risk factor for Parkinson's disease in Finnish and Greek populations (Clarimon et al., 2005). The discrepancy between these results remains to be elucidated.

The SNP in the 3′ non-coding region of *FGF20* can be a risk factor for Parkinson's disease. The risk allele disrupts a binding site for microRNA-433, increasing *FGF20* mRNA translation. This increase in *FGF20* mRNA translation has been correlated with increased α-*synuclein* expression. As α-synuclein is the principal component of filamentous Lewy bodies, the defining pathological hallmark of Parkinson's disease, these findings suggest a novel mechanism of action for the risk of Parkinson's disease (Wang et al., 2008). In addition, *FGF20* and α-*synuclein* were also shown to be associated with sporadic Parkinson's disease (Mizuta et al., 2008). However, no association between the SNP in *FGF20*, microRNA-433, or α-*synuclein* expression and Parkinson's disease have been reported (Wider et al., 2009; de Mena et al., 2010). The discrepancy between these results remains to be elucidated.

The genetic variability of the monoamine oxidase B gene (*MAOB*) has also been suggested as a risk factor for Parkinson's disease. Both FGF20 and MAOB are in the dopamine bio-pathway. SNP variants in *FGF20* and *MAOB* show evidence of statistical interactions, which emphasizes the importance of considering them jointly in the genetic analysis of Parkinson's disease, and illustrates the potential patterns of biological interactions contributing to the risk of Parkinson's disease (Gao et al., 2008).

A genome-wide association study (GWAS) to examine many common genetic variants was conducted in different individuals to identify any variant associated with a trait. The GWAS typically focused on associations between SNPs and traits such as major diseases. *FGF20* was shown to be a risk factor for Parkinson's disease by the GWAS (IPDGC, 2011; Pihlstrøm et al., 2013; Wang et al., 2013).

## FGF20 in the neural differentiation of stem cells into dopaminergic neurons

Neural stem (NS) cells are multipotent cells characterized by their capability to differentiate into neurons, astrocytes, and oligodendrocytes, and have been considered as cell resources for restorative transplantation strategies in Parkinson's disease. Nurr1 is a transcription factor of the thyroid hormone/retinoic acid nuclear receptor superfamily that is required for the induction of dopaminergic neurons. However, Nurr1 alone is not sufficient to induce a dopaminergic phenotype in NS cells. A co-culture of *Nurr1*-transfected NS cells with Schwann cells overexpressing *Fgf20* was shown to induce dopaminergic neurons in NS cells. Differentiated *Nurr1*-NS cells retained both neuronal morphology and tyrosine hydroxylase expression after transplantation into the striatum of 6-hydroxydopamine-lesioned rats. However, neuritogenesis was only observed after postnatal grafts. These results suggest that Fgf20 promotes the differentiation of *Nurr1*-NS cells into dopaminergic neurons and that additional factors are required for the efficient differentiation of dopaminergic neurons in the adult brain (Grothe et al., 2004).

Embryonic stem (ES) cells are pluripotent cells derived from the inner cell mass of the preimplantation blastocyst. These cells have many of the characteristics required of a cell source for cell-replacement therapy, including proliferation and differentiation capacities. ES cells are also promising donor cell sources for cell-replacement therapy in Parkinson's disease. FGF20 acts synergistically with FGF2 to increase the number of dopaminergic neurons in primate ES cell–derived neurospheres composed of neural progenitors. Dopaminergic neurons generated from primate ES cells were transplanted into 1-methyl-4-phenyl-1,2,3,6-tetrahydropyridine–treated (MPTP-treated) primates, a primate model for Parkinson's disease. Behavioral studies and functional imaging revealed that the transplanted dopaminergic cells functioned as dopaminergic neurons and attenuated MPTP-induced neurological symptoms (Takagi et al., 2005).

Parthenogenesis has attracted attention as an alternative method to derive ES cells that does not involve the destruction of viable embryos. Transplantation of dopaminergic neurons generated from parthenogenetic primate ES cells restored motor function in hemi-Parkinsonian, 6-hydroxy-dopamine-lesioned rats. Exposure to FGF20, along with WNT5a and FGF2, at the final stage of *in vitro* differentiation enhanced the maturation and *in vivo* survival of dopaminergic neurons and, correspondingly, the extent of motor recovery in transplanted animals (Sanchez-Pernaute et al., 2008). Induced pluripotent stem (iPS) cell-derived dopaminergic neurons were also shown to integrate into the striatum of Parkinsonian rats with behavioral improvements (Gibson et al., 2012). However, experiments on iPS cells using FGF20 have not been reported.

Neuronal differentiation in human ES cells was induced by co-culturing with PA6 mouse stromal cells. The number of tyrosine hydroxylase-expressing neurons significantly increased in culture medium supplemented with FGF20. Cultured cells also expressed other midbrain dopaminergic markers, which suggests that some differentiate into midbrain dopaminergic neurons. However, FGF20 has no effect on the size of the soma area or neurite length of dopaminergic neurons. FGF20 significantly reduced the proportion of cells undergoing cell death. These results indicate that FGF20 specifically increased the yield of dopaminergic neurons from human ES cells grown on PA6 feeder cells, and at least part of this effect was due to a reduction in cell death (Correia et al., 2007). In addition, FGF20 along with FGF2 enhanced dopaminergic neuron differentiation from human ES cell-derived neural progenitor cells directly without co-culturing with PA6 cells (Shimada et al., 2009).

## Conclusions

*Fgf20* is expressed in the SNpc of the midbrain. Fgf20 significantly enhances the survival of cultured dopaminergic neurons in a paracrine manner. In the rat model of Parkinson's disease, Fgf20 affords significant protection against the loss of dopaminergic neurons. The significant correlation of Parkinson's disease with SNPs within *FGF20* indicated that *FGF20* genetic variability is a risk factor for Parkinson's disease. Fgf20 promotes differentiation of cultured cells into dopaminergic neurons, and attenuated neurological symptoms in animal models of Parkinson's disease. These findings indicate the importance of FGF20 in both the differentiation and survival of dopaminergic neurons and the etiology and therapy of Parkinson's disease. Further studies on FGF20 will provide useful clues on the etiology and therapy of Parkinson's disease.

### Conflict of interest statement

The authors declare that the research was conducted in the absence of any commercial or financial relationships that could be construed as a potential conflict of interest.
